# Fabrication of Piezoelectric ZnO Nanowires Energy Harvester on Flexible Substrate Coated with Various Seed Layer Structures

**DOI:** 10.3390/nano11061433

**Published:** 2021-05-28

**Authors:** Taoufik Slimani Tlemcani, Camille Justeau, Kevin Nadaud, Daniel Alquier, Guylaine Poulin-Vittrant

**Affiliations:** GREMAN UMR 7347, Université de Tours, CNRS, INSA Centre Val de Loire, 37071 Tours, France; camille.justeau@univ-tours.fr (C.J.); kevin.nadaud@univ-tours.fr (K.N.); daniel.alquier@univ-tours.fr (D.A.); guylaine.poulin-vittrant@univ-tours.fr (G.P.-V.)

**Keywords:** ZnO nanowires, energy harvester, seed layer, flexible piezoelectric nanogenerator, hydrothermal synthesis

## Abstract

Flexible piezoelectric nanogenerators (PENGs) are very attractive for mechanical energy harvesting due to their high potential for realizing self-powered sensors and low-power electronics. In this paper, a PENG that is based on zinc oxide (ZnO) nanowires (NWs) is fabricated on flexible and transparent Polydimethylsiloxane (PDMS) substrate. The ZnO NWs were deposited on two different seed layer structures, i.e., gold (Au)/ZnO and tin-doped indium-oxide (ITO)/ZnO, using hydrothermal synthesis. Along with the structural and morphological analyses of ZnO NWs, the electrical characterization was also investigated for ZnO NWs-based flexible PENGs. In order to evaluate the suitability of the PENG device structure, the electrical output performance was studied. By applying a periodic mechanical force of 3 N, the ZnO NWs-based flexible PENG generated a maximum root mean square (RMS) voltage and average power of 2.7 V and 64 nW, respectively. Moreover, the comparison between the fabricated device performances shows that a higher electrical output can be obtained when ITO/ZnO seed layer structure is adopted. The proposed ZnO NWs-based PENG structure can provide a flexible and cost-effective device for supplying portable electronics.

## 1. Introduction

There has been a significant recent emphasis on mechanical energy harvesting for scavenging wasted or freely available ambient energy to generate electricity. In this context, piezoelectric nanogenerators (PENGs) are required to play a noteworthy role for long lifetime and reliable power supply [1,2].

During the last decade, many research studies have been implemented about the structural design, suitable material choices, understanding of the working mechanism, modeling, and performance optimization of the PENGs. Until recently, PENGs have been intensively developed and applied to harvest various forms of mechanical energies, starting with human motions to environmental scavenging [3,4]. Further, the adoption of flexible and stretchable PENGs has high research interest since it opens new opportunities for the development of flexible and wearable electronics [5,6]. Moreover, low-cost flexible substrates can be utilized for reducing production costs, which can make further progress on the mechanical energy harvesting market in terms of shortening the energy payback time. Besides low-cost flexible substrates, the use of low-cost materials is needed to obtain low production costs. Furthermore, ZnO, as a piezoelectric material, is beneficial for low cost as well as biosafety implementation, compared to lead-based PENGs [7].

ZnO is a promising material for a huge range of energy-saving applications, owing to its attractive semiconducting and piezoelectric properties [7,8]. Many reports have been already published on the physical properties of ZnO including nanorods (NRs) and nanowires (NWs) [9,10,11,12,13,14]. Ascribed to their remarkable properties, ZnO NWs have found widespread use in the development of PENGs.

In this work, we focus on the fabrication and characterization of ZnO NWs-based flexible PENGs with different seed layer structures. This report closely follows our previous work on rigid PENGs in which we reported the integration of seed layer to fabricate ZnO NWs-based rigid PENGs [11]. Herein, the growth of ZnO NWs using a low-temperature hydrothermal synthesis is reported. Furthermore, Au/ZnO and ITO/ZnO seed layer structures have been employed for the growth of ZnO NWs. Hence, in the present investigation, a proper and sincere attempt is made to understand the development of PENGs from the synthesis of ZnO NWs on flexible substrate right through to the design and fabrication of flexible PENG devices. The preparation of flexible substrates and the fabrication process of flexible PENGs are explained in detail in the Experimental Section.

## 2. Experimental

### 2.1. Flexible Substrate Preparation

Previous work from our group has already reported on the fabrication of PENGs by Dahiya et al. [15]. Originally, the PENGs were fabricated on a PDMS layer coated on a silicon substrate for easy handling during the fabrication steps. The PENGs were then released by peeling off the final devices from the Si substrate. However, Boubenia et al. [16] reported a decrease in the PENGs electrical performances after the peeling-off step of the device from the silicon substrate. The peeling-off step appears to introduce cracks and constraints in the metal layer electrode that could influence its durability and electrical performances; thus, this could lead to a decrease in the device lifetime.

**Figure 1:**
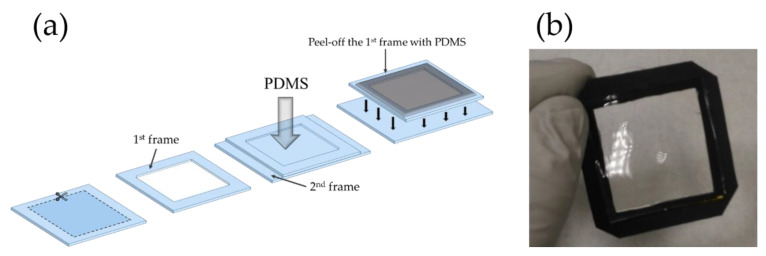
(**a**) Schematic illustration of PDMS substrate fabrication steps and (**b**) photograph of the PDMS substrate attached to the frame.

Therefore, in this paper, we present a new process for PDMS substrate fabrication that helps to keep the PENGs’ integrity before electrical measurements. The present proposed PENG process is different, compared with the previous device protocols [15,16,17]. Here, the complete PENG device was not lifted off from the rigid substrate, but only the PDMS layer was peeled off before starting the fabrication steps of the device. To avoid peeling off the entire PENG from the substrate, the PDMS film was only attached by its edges to a rigid frame. To do so, as described in Figure 1a, a rigid PET frame was placed on a plane PET surface. Then, a degassed mixture of elastomer and curing agent (10:1) forming liquid PDMS was spin-coated on the PET setup at 500 rpm for 15 s and then heated on a hot plate at 100 °C for 45 min. While curing, the PDMS was attached to the first PET frame; afterward, both elements were peeled off from the plane PET surface, freeing the PDMS surface (Figure 1b).

### 2.2. Bottom Electrode-Seed Layers Preparation (ITO/ZnO, Ti/Au/ZnO) and Growth of the ZnO Nanowires

In this study, two different bottom electrodes, i.e., Ti/Au and ITO, were deposited on the top of the PDMS substrate. The first electrode, Ti/Au (50 nm/100 nm), was DC sputtered at room temperature as follows: power of 500 W and pressure of 5 mTorr under argon atmosphere.

The second type of electrode consisted of coating the PDMS substrate with an ITO layer. In order to avoid cracks on its surface and ensure good conductivity, the ITO deposition protocol was based on the report of Lien et al. [18] and Casper et al. [19]. Therefore, a 400 nm thick layer of ITO was radio frequency (RF) sputtered at room temperature in two steps, as described in Table 1. The first thin ITO layer was sputtered at lower power to avoid any deformation of the PDMS substrate, which could impact the electrical integrity of ITO afterward. Once the PDMS substrate was prepared to receive the rest of the ITO layer, the second deposition step was performed at 65 W.

At last, on both electrode surfaces, a thin ZnO seed layer (100 nm) was deposited using RF sputtering. Specifically, the sputter power was 65 W, and the chamber pressure was 5 mTorr under Ar ambient. Therefore, to control the crystallinity and the morphology of ZnO NWs, the ZnO seed layer is required [20,21,22]. For the hydrothermal growth of ZnO NWs, the complete procedure was previously described in detail by Tlemcani et al. [10].

### 2.3. Fabrication of PENG Device

**Figure 2:**
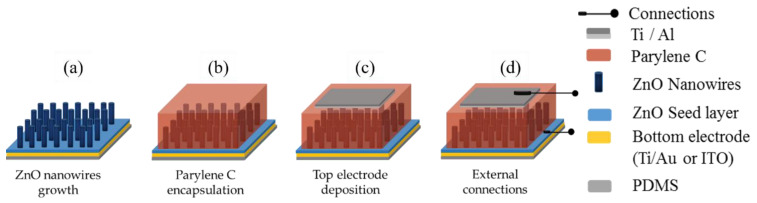
Fabrication steps of flexible PENG: (**a**) hydrothermal growth of ZnO NWs, (**b**) parylene-C deposition, (**c**) top electrode deposition, and (**d**) wires connection.

Figure 2 illustrates the fabrication process steps of the PENG device. ZnO NWs were synthesized on the PDMS substrate using the hydrothermal method, as shown in Figure 2a. Then, a parylene-C polymer matrix was deposited on the grown ZnO NWs by vapor phase pyrolysis technique following the Gorham steps [23], as seen in Figure 2b. In order to insulate the ZnO NWs from each other and create a capacitive coupling between the NWs and the top electrode, the parylene-C layer needed to be infiltrated into the sample [11,24,25].

Then, the surface of the parylene-C was masked to deposit a 1.2 cm^2^ Ti/Al (100 nm/400 nm) layer by evaporation (Figure 2c).

**Figure 3:**
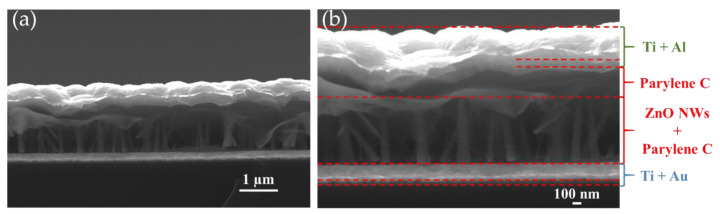
Cross-sectional SEM images of PENG at (**a**) 15,000× and (**b**) 30,000× magnifications.

An example of cross-sectional scanning electron microscopy (SEM) images of the PENG can be seen in Figure 3. It clearly shows the good infiltration of parylene-C between the ZnO NWs.

**Figure 4:**
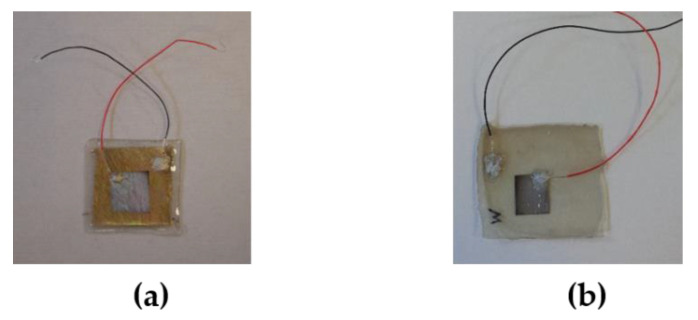
Photograph of PENGs devices made from ZnO NWs deposited on (**a**) Au/ZnO and (**b**) ITO/ZnO seed layer structures.

Finally, the silver conductive adhesive paste was used to connect copper wires to the top and the bottom electrodes of the harvester (Figure 2d), and the whole PENG was encapsulated in PDMS to protect the devices from external factors and to improve their durability during the electrical analyses. Once the entire PENG fabrication was completed, the PET frame was removed to allow flexibility. The visual aspect of the PENGs is shown in Figure 4.

### 2.4. Characterization Techniques

**Figure 5:**
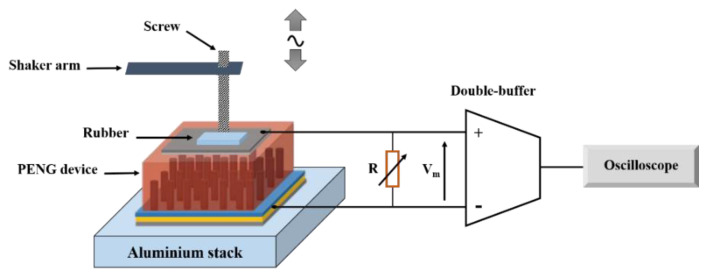
Test bench for PENG performance measurement.

The crystal structure of ZnO NWs was examined using X-ray diffraction (XRD, Brucker AXS D8 discover) operated with CuKα radiation. The surface morphology was observed using scanning electron microscopy (SEM, JEOL JSM-7900F). The extraction of the morphological parameters was conducted using Image J software on SEM images [10]. The electrical measurements were performed using the test bench designed and described in detail by our team [17], as shown in Figure 5.

The PENG was being solicited using the aluminum mechanical shaker, which impacted its active surface area (1 cm^2^). The device was also connected to a double-buffer circuit comprising a differential amplifier, which measured the output performances without making any parasitic bias and allowed us to use a resistive load higher than the input impedance of the oscilloscope [26].

## 3. Results and Discussion

**Figure 6:**
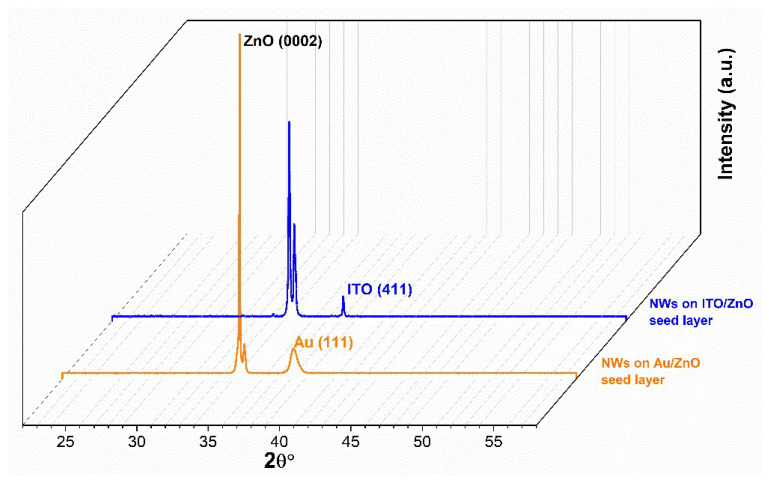
XRD 3D spectra of the ZnO NWs deposited on Au/ZnO and ITO/ZnO seed layer structures.

Figure 6 shows the XRD spectra of ZnO NWs deposited on Au/ZnO and ITO/ZnO seed layer structures, exhibiting the main diffraction peak of hexagonal würtzite ZnO structure (JCPDS Card No. 05-0664), without detection of any impurity phase. Therefore, ZnO NWs grown by hydrothermal technique led to a pure-phase material. The entire samples exhibit a strong peak at 34.4° corresponding to the lattice plane (0002) of würtzite ZnO structure, confirming that the NWs grew predominantly along the [0001] direction.

Additionally, it can be observed that the XRD pattern of ZnO NWs deposited on Au/ZnO presents the highest intensity of the (0002) peak, which is attributed to the improvement in the crystallinity of the NWs. Additionally, XRD patterns show a (0002) peak splitting for both samples. This can be related to the large lattice mismatch between seed layers and NWs, as detailed in our previous report [10].

**Figure 7:**
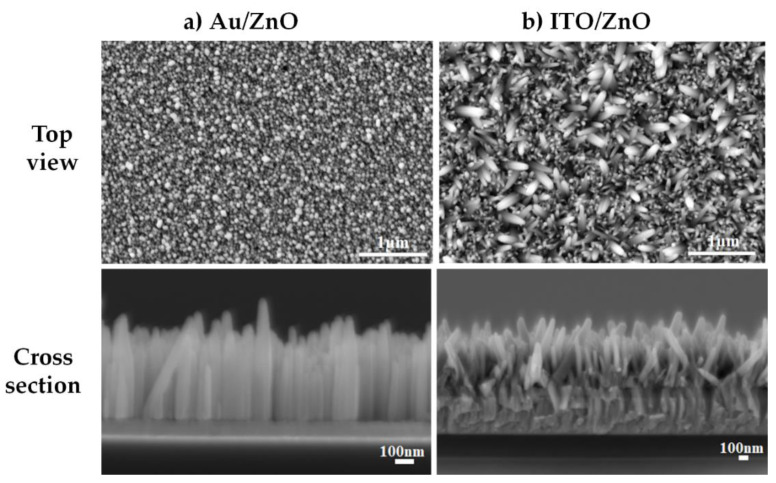
Top and cross-sectional views of SEM micrographs of ZnO NWs deposited on (**a**) Au/ZnO and (**b**) ITO/ZnO seed layer structures.

Top and cross-sectional views of SEM micrographs of ZnO NWs deposited on the two seed layers are shown in Figure 7. It is obviously observed that the alignment and morphology of the NWs are strongly influenced by the seed layer types. Moreover, the SEM micrographs show that the ZnO NWs deposited on Au/ZnO seed layer structure have a much better alignment (Figure 7a) with a perpendicular orientation to the substrate surface, whereas the ZnO NWs deposited on ITO/ZnO seed layer structure are randomly oriented.

This result is consistent with the XRD patterns, shown in Figure 6, in which it was seen that the NWs deposited on Au/ZnO seed layer structure showed better crystallinity than those on the ITO/ZnO one. If the seed layer is a crucial structure for the preparation of high-quality ZnO NWs, it is also known that catalysts such as Cu, Ag, and Au integrated on the ZnO seed layer enhance the growth quality of ZnO NWs [27,28]. All these results are in good accordance with our previous studies [10,11] and validate the fact that the seed layer structure has an important influence on the structural and morphological characteristics of the resulting ZnO NWs. Additionally, Table 2 shows the morphological characteristics of ZnO NWs deposited on different seed layer structures. It can be noticed that the length of the NWs with ITO/ZnO seed layer structure is larger than those with Au/ZnO one. As a general observation, the morphological values of the NWs, particularly the density and diameter related to the two types of seed layer structures, are quite similar to each other.

**Figure 8:**
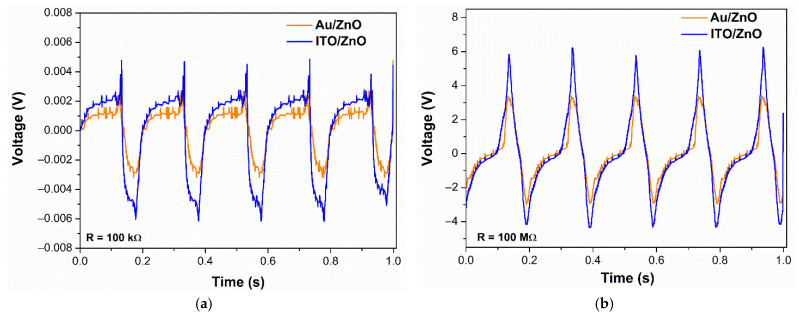
Voltage amplitude of the fabricated PENGs with different seed layer structures at a load resistance of (**a**) 100 kΩ and (**b**) 100 MΩ, when applying an alternative mechanical excitation with a magnitude of 3 N at a frequency of 5 Hz.

The NWs grown on different seed layer structures have been used to realize PENGs, as described in Section 2.2. Those PENGs have been characterized by applying an alternative mechanical excitation with a magnitude of 3 N at a frequency of 5 Hz. The resistive load is connected at the output of the PENGs to emulate the current consumption of an external circuit, and this resistive load has been varied from 100 kΩ to 100 MΩ. Furthermore, the voltage at the resistive load is measured using an oscilloscope through the double buffer circuit [26]. Figure 8 shows the measured voltage at the output of the PENGs for two values of the resistive load, 100 kΩ (Figure 8a) and 100 MΩ (Figure 8b). It is clear that the resistive load value strongly affects the output voltage. We note that the curves are quite similar for both samples while, in each case, the voltage magnitude is in the mV range for 100 kΩ, and in the V range for 100 MΩ.

**Figure 9:**
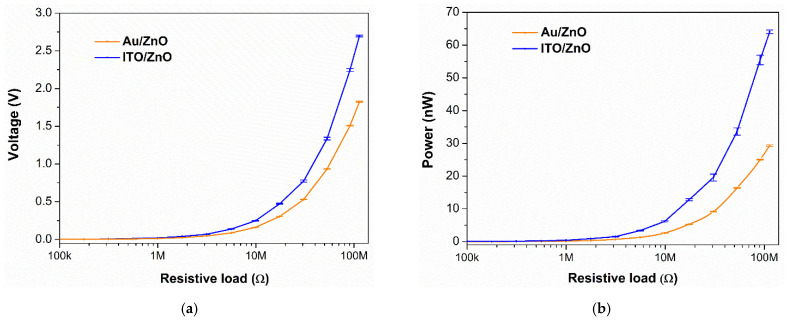
RMS output voltage (**a**) and average power (**b**) generated by PENGs with different seed layer structures. Measured RMS voltage and average power for a load resistance ranging from 100 kΩ to 100 MΩ, when applying an alternative mechanical excitation with a magnitude of 3 N at a frequency of 5 Hz.

Using the output voltage curves, the average electrical power was computed as a function of the load resistance, as reported in Figure 9 [26]. The root mean square (RMS) output voltage and the average power increase when the resistive load increases and the curves do not present a maximum on this range of resistive load, as generally observed, indicating that the optimal load can be certainly higher than 100 MΩ. In this case, the PENG can be used in two ways, either to charge a capacitor [29] or as an energy-autonomous wearable sensor [30].

The sample with the ITO/ZnO seed layer structure shows a maximum average power and RMS voltage of 64 nW and 2.7 V, respectively, which are higher than the values of the sample with Au/ZnO seed layer. The relatively low performance of the PENG device with Au layer (metal electrode) may be attributed to the interface adhesion deterioration, leading to possible delamination, wrinkling, or even cracking of the stacked film layers caused by the applied mechanical stress. It is important to note that the adherence of metal electrodes to PDMS substrate is usually poor [31]. Furthermore, among materials generally used in electronics devices, a thin metal layer is one of the weakest parts against mechanical stress and has a potential problem of cracking when stressed because of the degradation of its morphological and electrical properties during repeated mechanical excitation [32,33,34,35]. This could result in bad electrical charge transfer and increase the electrical losses, preventing the metal layer from being a functional bottom electrode. Moreover, the difference in the PENG performances might be explained by the different morphological characteristics of ZnO NWs. Actually, when comparing the aspect ratio, determined by the ratio of length over diameter (Table 2), the NWs deposited on Au/ZnO exhibited the lowest value with 9, compared to a ratio of 10 for NWs deposited on the ITO/ZnO seed layer. This reveals that the better uniformity obtained for NWs deposited on Au/ZnO has also promoted a decrease of their aspect ratio, which is needed to be high enough to afford large deformation of the NWs and to achieve efficient electromechanical energy conversion [36]. The electrical performances such as maximum peak instantaneous voltage (*V*_peak_), maximum RMS voltage (*V*_RMS_) _max_, maximum RMS short circuit current (*I*_sc_), and maximum average power (*P*_av_) _max_ of both samples, deduced from Figure 9a,b, are summarized in Table 3. In this table, the electrical energy $(W=P_{\mathrm{av}}/f$) generated by each PENG is also indicated, where *f* is the frequency value of the applied mechanical force (here *f* = 5 Hz).

To obtain a further insight into the performances of ZnO NWs-based PENGs, Table 4 shows a comparison of the obtained PENG device performances with our previously reported values and some of the interesting studies available in the literature. These reports have been devoted to the fabrication of PENG devices on a rigid silicon substrate via Au/ZnO seed layer (*V*_peak_ of 0.27 V) [11], flexible PDMS substrate via Au/ZnO seed layer (*V*_peak_ of 9.1 V [17], *V*_peak_ of 2.03 V [37]), and flexible PDMS substrate using ITO/ZnO seed layer (*V*_peak_ of 8 V [38]). It is evident from Table 4 that the value of the applied stress (mechanical loading) was not always mentioned, which is essential to compare the characteristics among the PENG devices. A notable performance has been achieved by Dahiya et al. for flexible devices. However, it can be readily observed that the mechanical stress (13 N) was four times higher, compared with the present study. Additionally, the proposed flexible PENG employs a transparent ITO electrode layer. Further comparisons with the state-of-the-art performances for ZnO NWs-based PENG devices are not easy due to the lack of a standardized testing protocol as well as insufficient information available regarding the experimental protocol in the literature.

Consequently, the difference between the performances of flexible and rigid devices might be related to several factors. An explanation is that the transition from a rigid to a flexible substrate can probably modify the way in which the NWs are constrained, and the distribution of the forces can be more three-dimensional than only along one direction (vertical) [39].

Overall, the presently proposed ZnO NWs-based flexible PENGs have shown quite interesting performances. Further thorough investigations when varying the thickness of both the seed layer structure and the PDMS substrate, and/or adoption of aluminum-doped ZnO (AZO) instead of ZnO seed layer could further increase the device performance levels.

## 4. Conclusions

In summary, flexible PENGs based on ZnO NWs have been fabricated employing Au/ZnO and ITO/ZnO seed layer structures. This approach involves a new protocol for the preparation of PDMS substrate with an original design of functional flexible PENG devices. These devices are based on the deposition of ZnO NWs on PDMS substrate using different seed layer structures exhibiting high crystallinity, good flexibility, and promising electrical properties. The fabricated devices with ITO/ZnO seed layer structure showed twofold higher average power than those with the Au/ZnO one. This suggests that it is possible to employ transparent and cost-effective ITO electrodes to fabricate energy-efficient flexible PENG devices. However, though this study presents a new way to fabricate flexible PENGs, exhibiting a promising prospect for transparent flexible devices, further progresses and challenges are still needed to improve the substrate flexibility, fabrication process, and electrical performances.

## Figures and Tables

**Table 1:** ITO sputtering conditions.

Step	Pressure	Gas Flow	Power	Deposition Time	Thickness
1	5 mTorr	O_2_:Ar (1:1)	50 W	5 min	≈20 nm
2	5 mTorr	Ar	65 W	58 min	380 nm

**Table 2:** Morphological characteristics of ZnO NWs deposited on Au/ZnO and ITO/ZnO seed layer structures.

Seed Layer Structure	NWs Length (µm)	NWs Diameter (µm)	Density (NWs/µm^2^)	NWs Aspect Ratio
Au/ZnO	0.59 ± 0.15	0.07 ± 0.01	39.2 ± 0.3	9
ITO/ZnO	0.70 ± 0.17	0.07 ± 0.02	41.4 ± 0.2	10

**Table 3:** PENG device performances for various seed layer structures: *V*_peak_, *V*_RMS (max)_, *I*_sc (RMS)_, *P*_av (max)_, energy during one period of mechanical input force (*W*), and corresponding optimal load (*R*_opt_), when applying an alternative mechanical excitation with a magnitude of 3 N at a frequency of 5 Hz.

Seed Layer Structure	*V*_peak_ (V)	*V*_RMS (max)_ (V)	*I*_sc (RMS)_ (nA)	*P*_av (max)_ (nW)	*W* (nJ)	*R*_opt_ (MΩ)
Au/ZnO	3.9	1.8	15	29	5.8	>100
ITO/ZnO	6.8	2.7	28	64	12.8	>100

**Table 4:** Comparison of the characteristics of ZnO NWs-based PENG devices fabricated on different substrates and seed layers.

Substrate	Seed Layer Structure	*V*_peak_ (V)	Mechanical Loading: Force (N)	Device Dimensions (cm^2^)	Ref.
Silicon	Au/ZnO	0.27	3	1.2	[11]
PDMS	Au/ZnO	9.1	13	1.2	[17]
PDMS	Au/ZnO	2.03	N/A	1	[37]
PDMS	ITO/ZnO	8	N/A	1.5	[38]
PDMS	ITO/ZnO	6.8	3	1.2	This work

## Data Availability

The data presented in this study are available from the corresponding author upon request.
